# Effectiveness of Whole-Body High-Intensity Interval Training on Health-Related Fitness: A Systematic Review and Meta-Analysis

**DOI:** 10.3390/ijerph19159559

**Published:** 2022-08-03

**Authors:** Corentin Scoubeau, Bruno Bonnechère, Miriam Cnop, Vitalie Faoro, Malgorzata Klass

**Affiliations:** 1 Cardio-Pulmonary Exercise Laboratory, Faculty of Motor Sciences, Université Libre de Bruxelles, 1070 Bruxelles, Belgium; corentin.scoubeau@ulb.be (C.S.); vitalie.faoro@ulb.be (V.F.); 2 REVAL Rehabilitation Research Center, Faculty of Rehabilitation Sciences, Hasselt University, 3590 Diepenbeek, Belgium; bruno.bonnechere@uhasselt.be; 3 ULB Center for Diabetes Research, Université Libre de Bruxelles, 1070 Bruxelles, Belgium; miriam.cnop@ulb.be; 4 Division of Endocrinology, Erasmus Hospital, Université Libre de Bruxelles, 1070 Bruxelles, Belgium; 5 Research Unit in Biometry and Exercise Nutrition, Faculty of Motor Sciences, Université Libre de Bruxelles, 1070 Bruxelles, Belgium; 6 Laboratory of Applied Biology, Research Unit in Applied Neurophysiology, Faculty of Motor Sciences, ULB Neuroscience Institute, Université Libre de Bruxelles, 1070 Bruxelles, Belgium

**Keywords:** functional high-intensity training, body composition, musculoskeletal fitness, cardiorespiratory endurance, metabolic risk factors

## Abstract

Due to its versatility, whole-body high-intensity interval training (WB-HIIT) can be proposed to the general population and patients to improve health-related fitness. However, its effectiveness compared to traditional aerobic continuous or interval trainings has yet to be determined. A search of four electronic databases was conducted. Studies reporting the effects of WB-HIIT on cardiorespiratory fitness (CRF), fat mass, fat-free mass, musculoskeletal fitness and metabolic risk factors were included. Standardized mean differences (SMD) between WB-HIIT and no exercise or traditional aerobic trainings were calculated. A meta-regression assessed the effect of total training time on the different outcomes. Twenty-two studies were included in the systematic review and nineteen in the meta-analysis. Compared to no exercise, WB-HIIT improves CRF (SMD: 0.75; 95%CI: 0.28, 1.23; *p* < 0.001), fat-free mass (SMD: 0.38; 95%CI: 0.11, 0.65; *p* < 0.001), fat mass (SMD: 0.40; 95%CI: 0.09, 0.72; *p* < 0.001) and musculoskeletal fitness (SMD: 0.84; 95%CI: 0.61, 1.08; *p* < 0.001). Compared to other aerobic trainings, WB-HIIT has a lower effect on CRF (SMD: −0.40; 95%CI: −0.70, −0.11; *p* = 0.007), a similar effect on fat-free mass (SMD: −0.04; 95%CI: −0.44, 0.35; *p* = 0.8) and fat mass (SMD: −0.07; 95%CI: −0.39, 0.25; *p* = 0.7), and a larger effect on musculoskeletal fitness (SMD: 0.42; 95%CI: 0.14, 0.71; *p* = 0.003). WB-HIIT overall effect and specific effect on CRF and fat mass were associated with total training time. The systematic review did not provide evidence of metabolic risk improvement. Despite a slightly lower effect on CRF, WB-HIIT is equally effective as traditional aerobic trainings to improve body composition and more effective to enhance musculoskeletal fitness, which is essential for execution of daily tasks.

## 1. Introduction

Among the barriers to physical activity, environmental context, cost, access to gym facilities, lack of time or bad weather are often mentioned [1]. Alternatives to traditional forms of training that can be performed anywhere, incur low cost and are adaptable to different fitness levels are therefore needed. Recently, high-intensity functional training has become popular in the general population and among athletes, but also in rehabilitation settings due to its versatility and accessibility [2,3]. Similarly to traditional high-intensity interval training (HIIT) [4], high-intensity functional training alternates high-intensity bouts with periods of recovery, but instead of biking, running or rowing, it uses multi-joint aerobic and strengthening exercises that are closer to athletic and daily living movements [2,5].

High-intensity functional training protocols consist of: (1) whole-body high-intensity interval training (WB-HIIT) using only the body weight as resistance (e.g., squats, push-ups, mountain climbers, etc.) and (2) weightlifting exercises (e.g., snatch, shoulder press, deadlift, etc.) [2]. WB-HIIT is easily adaptable to different fitness levels [6] and presents a lower risk of injury as compared to higher load weightlifting exercises [7]. It can therefore be proposed to a larger audience. In addition, due to its multimodal nature, it has the potential to improve several fitness components [2].

Despite these advantages, the actual effectiveness of WB-HIIT to improve health-related fitness, especially when compared to traditional forms of aerobic training, has yet to be determined. Traditional aerobic forms of training include HIIT and moderate- or vigorous-intensity continuous training (MICT or VICT) in which intensity remains constant. MICT is generally performed at an intensity between 64% and 77% of maximal heart rate (HRmax), while in VICT, intensity exceeds 77% of HRmax [8].

In 2020, a meta-analysis compared the effect of WB-HIIT to MICT and HIIT on CRF in healthy individuals [5]. The aim of the present systematic review and meta-analysis was to include other important health-related fitness components (i.e., fat mass, fat-free mass, musculoskeletal fitness and metabolic health) to the comparison between WB-HIIT and traditional aerobic trainings or no exercise, and to enlarge the investigated populations by including trials on participants with metabolic risk factors or physical limitations. Since WB-HIIT studies include a wide variety of exercise protocols differing in duration and frequency, we also aimed to evaluate the impact of total training time on the effects.

## 2. Methods

### 2.1. Search Strategy

This review was prospectively registered with PROSPERO (registration number: CRD42021266221). The Preferred Reporting Items for Systematic Reviews and Meta-Analyses (PRISMA) provided support in guiding this review [9].

Potentially relevant studies were identified from four databases (PubMed, Scopus, Embase and ScienceDirect) up to March 2022. MeSH terms and free words referring to different training modalities were used (“high-intensity functional training”; “Tabata training”; “whole body” AND “high intensity” AND training; functional AND “circuit training”; “whole body” AND “circuit training”). References from selected papers and from other relevant articles were screened for additional studies in accordance with the snowball principle. The search was limited to peer-reviewed journal articles published in English.

### 2.2. Study Selection

Only randomized controlled trials on humans with a longitudinal design were eligible for inclusion. Animal studies, abstracts, case reports, study protocols, reviews or trials investigating acute effects of training were excluded. To be included, studies had to use a WB-HIIT intervention without diet modification or supplementation and test at least one of the following outcomes: CRF, fat mass, fat-free mass, musculoskeletal fitness or metabolic risk factors [10]. Only WB-HIIT interventions using body weight as resistance and multi-joint exercises where subjects were instructed to perform as many repetitions as possible, leading to an important increase in heart rate during the exercise phases, were considered. The use of small equipment (e.g., TRX^®^, step, elastic band, etc.) was accepted, but not the use of weightlifting exercises. Thus, protocols using CrossFit, heavy weight bearing, Olympic weightlifting, and functional training combined with aerobic training or strength training were excluded. This choice was made for the following reasons: the focus on health-related fitness, the accessibility of WB-HIIT to a large audience and in various settings, and to facilitate comparison with traditional aerobic trainings. Trials in which the training intervention was not defined or in which investigators did not design the exercise program were excluded. Studies had to include a comparator group, either traditional aerobic training (HIIT, MICT or VICT) or no exercise. Studies using a comparator group of combined training (e.g., aerobic plus strength training) or comparing different forms of WB-HIIT without any other comparator group were excluded.

Articles found in the different databases were collated using Endnote X9. Duplicates were removed by the software and verified by a reviewer. A first selection was made based on title and abstract and then on the full text by two reviewers.

### 2.3. Data Extraction

To investigate health-related fitness components, data related to CRF (maximal oxygen uptake (VO_2_max)), total fat mass, fat-free mass (including lean mass and muscle mass), musculoskeletal fitness (muscle strength and endurance) and metabolic risk factors (high- and low-density lipoproteins (HDL and LDL) cholesterol levels, fasting plasma glucose, visceral fat and blood pressure at rest) were extracted. Detailed data extraction was undertaken independently by two researchers and encoded in an Excel sheet including first author’s name, publication year, title, population, exercise and control groups, number and age of the participants, male/female ratio, outcomes and related results. When two papers were published [11,12,13,14] with the same participants but different outcomes, the participants were counted only once.

### 2.4. Quality Assessment and Risk of Bias

Quality of studies was assessed using the PEDro scale. Two researchers evaluated the articles independently. Discrepancies were arbitrated by a third person. To detect potential publication bias, funnel plot analysis and Egger’s test for the intercept was applied to check the asymmetry. Sensitivity analysis was performed using a leave-one-out method.

### 2.5. Data Synthesis and Statistical Analysis

For each component, a minimum of three studies was required to be included in the meta-analysis [15], comparing either WB-HIIT to no exercise or other training types. The following outcomes were included in the meta-analysis: VO_2_max for CRF, total fat mass and fat-free mass, and performance at muscle strength or endurance tests for musculoskeletal fitness. Since only two studies investigated metabolic risk factors for WB-HIIT compared to no exercise and two for WB-HIIT compared to traditional forms of aerobic training, this component was included in the systematic review but not in the meta-analysis. For the meta-analysis, mean pre- to post-changes and standard deviation were calculated. Standardized mean difference (SMD) and 95% CI were calculated for statistical analysis. SMD was defined as the between group difference in mean values divided by the pooled SD computed using the Hedge’s g method. Heterogeneity between the studies was assessed using I^2^. If the required data were not available in numeric text, authors were contacted to obtain the numerical data. If data could not be provided, the study was excluded from the meta-analysis. Data were then pooled using random-effect meta-analysis with subgroup analyses to evaluate the effect on each component: CRF, fat-free mass, fat mass and musculoskeletal fitness. A fixed-effect model was then performed to assess the overall effect of training on the different health-related fitness components. This model was chosen to give the same weight to each component, regardless of the number of individual observations. A first analysis was made between WB-HIIT and no exercise and a second one between WB-HIIT and traditional aerobic trainings (HIIT, MICT and VICT).

For studies comparing WB-HIIT to no exercise, random-effects meta-regression analyses were computed to assess the association between the magnitude of effects (overall and for CRF, fat-free mass, fat mass and musculoskeletal fitness) and total training time. Studies were weighted by the inverse of the sum of within- and between-study variance. Total training time was calculated by multiplying session duration by training frequency and by the number of weeks (Table 1). Since not all studies provided information about warm-up and cool-down duration, only the conditioning phase of the session was considered for training time calculation. Statistical analyses were performed at an overall significance level of 0.05. Statistics were conducted in RStudio (Rstudio Team, Boston, MA, USA, version 1.2.5042) with R (version 3.6.3).

## 3. Results

### 3.1. Search Results

**Figure 1:**
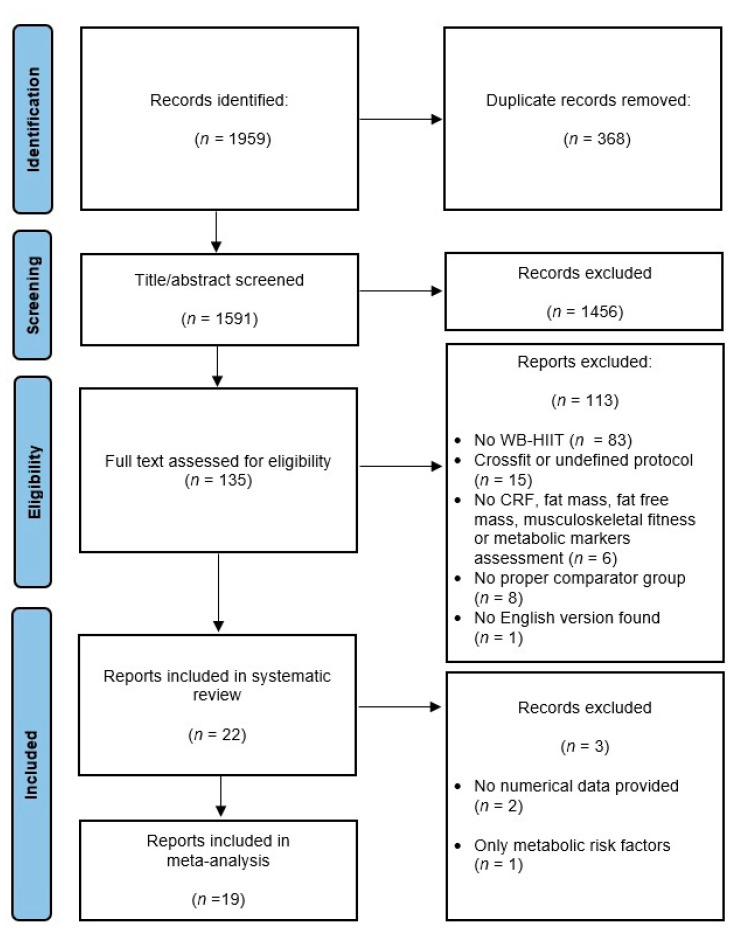
Flow diagram of the identification and screening procedure. WB-HIIT, whole-body high-intensity interval training; CRF, cardiorespiratory fitness.

Of the 1959 trials retrieved from databases, twenty-two studies were selected for the systematic review and meta-analysis. Two studies [16,17] were excluded from the meta-analysis since numerical data were not available and not provided by the authors. One study [18] was excluded since the only investigated component was metabolic risk factors. The meta-analysis hence includes nineteen studies representing a total of 657 participants (306 WB-HIIT, 155 traditional aerobic training, 196 no exercise). The flow chart of the study selection process is shown in Figure 1.

Characteristics of included studies are presented in Table 1 and narrative description of the intragroup changes after the interventions in Table 2. Results from the three excluded studies [16,17,18] were also included, as they are part of the systematic review.

### 3.2. Quality Assessment and Risk of Bias

Seventeen of the twenty-two initially selected studies had a PEDro score between 6 and 7, considered as “good” [19]. Four had a score of 5 and one had a score of 4, considered as “fair”. Except for one study that had blinded investigators [20], blinding was absent. Concealed allocation of participants was reported in only two studies [18,21]. Complete PEDro evaluation of the studies is shown in Table 3.

Funnel plot analysis did not reveal any significant asymmetry, regardless of the comparisons (WB-HIIT vs. no exercise: *p* = 0.054; WB-HIIT vs. traditional: *p* = 0.58), suggesting that there is no publication bias (see Supplementary Figures S1 and S2).

Sensitivity analyses were performed for the two comparisons and did not reveal change in the results of the meta-analysis after removal of any one study (see Supplementary Figures S3 and S4).

**Table 1:** Characteristics of the included studies.

Study	Population	Groups	Interventions	Exercise Intervention Protocols				Outcomes
				Session Duration (min)	IT Work/Rest Ratio	Frequency	Total Training Time (min)	
**Healthy participants**								
Ballesta-Garcia, 2019 [11]	Healthy adults	WB-HIIT vs. WB-MIIT vs. CTL	WB-HIIT: Whole-body exercises at high RPEs WB-MIIT: Whole-body exercises at moderate RPE CTL: No exercise	WB-HIIT: 18–40 WB-MIIT: 18–50	WB-HIIT: 60–90″/120–150″	WB-HIIT: 16 wks, 2×/wk WB-MIIT: 16 wks, 2×/wk	WB-HIIT: 928 WB-MIIT: 1088	MSF
Ballesta-Garcia, 2020 [12]	Healthy adults	WB-HIIT vs. WB-MIIT vs. CTL	WB-HIIT: Whole-body exercises at high RPEs WB-MIIT: Whole-body exercises at moderate RPE CTL: No exercise	WB-HIIT: 18–40 WB-MIIT: 18–50	WB-HIIT: 60–90″/120–150″	WB-HIIT: 16 wks, 2×/wk WB-MIIT: 16 wks, 2×/wk	WB-HIIT: 928 WB-MIIT: 1088	CRF
Blackwell et al., 2017 [20]	Healthy adults	WB-HIIT vs. HIIT	WB-HIIT: Whole-body exercises HIIT: Cycling at 95–110% Wmax	WB-HIIT: 11 HIIT: 11	WB-HIIT: 60″/90″ HIIT: 60″/90″	WB-HIIT: 4 wks, 3×/wk HIIT: 4 wks, 3×/w	WB-HIIT: 132 HIIT: 132	CRF, Metabolic risk factors
* Connolly et al., 2020 [18]	Healthy adults	WB-HIIT vs. CTL	WB-HIIT: Whole-body exercises CTL: No exercise	WB-HIIT: 15	WB-HIIT: 30″ low/20″ moderate/10″ high intensity	WB-HIIT: 12 wks, 3×/wk	WB-HIIT: 540	Metabolic risk factors
Engel et al., 2019 [22]	Healthy adults	WB-HIIT vs. CTL	WB-HIIT: Whole-body exercises with suspension trainer CTL: No exercise	WB-HIIT: 30	WB-HIIT: 20″/10″	WB-HIIT: 8 wks, 2×/wk	WB-HIIT: 480	MSF, Metabolic risk factors
Evangelista et al., 2019 [23]	Healthy adults	WB-HIIT vs. VICT	WB-HIIT: Whole-body exercises VICT: Running at 80% HRmax	WB-HIIT: 20 VICT: 20	WB-HIIT: 30″/30″	WB-HIIT: 6 wks, 3×/wk VICT: 6 wks, 3×/wk	WB-HIIT: 360 VICT: 360	Fat mass, Fat-free mass, MSF
Evangelista et al., 2021 [24]	Healthy adults	WB-HIIT vs. CTL	WB-HIIT: Whole-body exercises	WB-HIIT: 21	WB-HIIT: 40″/20″	Wb-HIIT: 6 wks, 3×/wk	WB-HIIT: 378	CRF, MSF
Islam et al., 2019 [25]	Healthy adults	WB-HIIT vs. VICT vs. CTL	WB-HIIT: Whole-body exercises VICT: Running at 85% HRmax CTL: No exercise	WB-HIIT: 4 VICT: 30	WB-HIIT: 20″/10″	WB-HIIT: 4 wks, 4×/wk VICT: 4 wks, 4×/wk	WB-HIIT: 60 VICT: 480	CRF, MSF
Jimenez-Garcia et al., 2019 [21]	Healthy adults	WB-HIIT vs. WB-MIIT vs. CTL	WB-HIIT: Squats using TRX^®^ at 90–95% HRmax WB-MIIT: Squats using TRX^®^ at 50–70% HRmax CTL: No exercise	WB-HIIT: 25 WB-MIIT: 25	WB-HIIT: 4′/3′ WB-MIIT: 4′/3′	WB-HIIT: 12 wks, 2×/wk WB-MIIT: 12 wks, 2×/wk	WB-HIIT: 600 WB-MIIT: 600	Fat mass, Fat-free mass, MSF
Lu et al., 2021 [26]	Healthy adults	WB-HIIT vs. HIIT	WB-HIIT: Whole-body exercises HIIT: Running	WB-HIIT: 4 HIIT: 4	WB-HIIT: 20″/10″ HIIT:30″/30″	WB-HIIT: 12 wks, 3×/wk HIIT: 12 wks, 3×/wk	WB-HIIT: 144 HIIT: 144	CRF, Fat mass, Fat-free mass, MSF
* McRae et al., 2012 [16]	Healthy adults	WB-HIIT vs. VICT vs. CTL	WB-HIIT: Whole-body exercises CT: Running at 85% HRmax CTL: No exercise	WB-HIIT: 4 VICT: 30	WB-HIIT: 20″/10″	WB-HIIT: 4 wks, 4×/wk CT: 4 wks, 4×/wk	WB-HIIT: 48 CT: 480	CRF, MSF
Menz et al., 2019 [27]	Healthy adults	WB-HIIT vs. HIIT	WB-HIIT: Whole-body exercises HIIT: Running	WB-HIIT: 12–16 HIIT: 12–16	WB-HIIT: 20″/10″ HIIT: 20″/10″	WB-HIIT: 4 wks, 3–4×/wk HIIT: 4 wks, 3–4×/wk	WB-HIIT: 191 HIIT: 191	CRF, Fat mass, Fat-free mass, MSF
Micielska et al., 2019 [28]	Healthy adults	WB-HIIT vs. CTL	WB-HIIT: Whole-body exercises CTL: Whole-body exercises	WB-HIIT: 25	WB-HIIT: 30″/10″	WB-HIIT: 5 wks, 3×/wk CTL: 2 sessions	WB-HIIT: 325 CTL: 50	CRF, Fat mass, Fat-free mass
Murawska-Cialowicz et al., 2020 [29]	Healthy adults	WB-HIIT vs. CTL	WB-HIIT: Whole-body exercises CTL: No exercise	WB-HIIT: 40	WB-HIIT: 20″/10″	WB-HIIT: 8 wks, 2×/wk	WB-HIIT: 640	CRF, Fat mass, Fat-free mass
Schaun et al., 2018 [13]	Healthy adults	WB-HIIT vs. HIIT vs. VICT	WB-HIIT: Whole-body exercises HIIT: Running at 130% VO_2_ max VICT: Running at 90–95% VT2	WB-HIIT: 8 HIIT: 8 VICT: 30	WB-HIIT: 20″/10″ HIIT: 20″/10″	WB-HIIT: 16 wks, 3×/wk HIIT: 16 wks, 3×/wk VICT: 16 wks, 3×/wk	WB-HIIT: 384 HIIT: 384 VICT: 1440	CRF, Fat mass
Schaun et al., 2019 [14]	Healthy adults	WB-HIIT vs. HIIT vs. VICT	WB-HIIT: Whole-body exercises HIIT: Running at 130% VO_2_ max) VICT: Running at 90–95% VT2	WB-HIIT: 8 HIIT: 8 VICT: 30	WB-HIIT: 20″/10″ HIIT: 20″/10″	WB-HIIT: 16 wks, 3×/wk HIIT: 16 wks, 3×/wk VICT: 16 wks, 3×/wk	WB-HIIT: 384 HIIT: 384 VICT: 1440	MSF
Schmidt et al., 2016 [30]	Healthy adults	WB-HIIT vs. CTL	WB-HIIT: Whole-body exercises CTL: No exercise	WB-HIIT 7: 1 × 7 WB-HIIT 14: 1 × 7 (wk 1–4), 2 × 7 (wk 5–8)	WB-HIIT: 30″/10″	WB-HIIT 7: 8 wks, 3×/wk WB-HIIT 14: 8 wks, 3×/wk	WB-HIIT 7: 168 WB-HIIT 14: 252	CRF, Fat mass, MSF
Sperlich et al., 2018 [31]	Healthy adults	WB-HIIT LV vs. WB-HIIT HV vs. CTL	WB-HIIT LV: Whole-body exercises (1×/day) WB-HIIT HV: Whole-body exercises (2×/day) CTL: No exercise	WB-HIIT LV: 1 × 6 WB-HIIT HV: 2 × 6	WB-HIIT LV: Not specified (6 min circuit) WB-HIIT HV: Not specified (6 min circuit)	WB-HIIT LV: 4 wks, 7×/wk WB-HIIT HV: 4 wks, 14×/wk	WB-HIIT LV: 168 WB-HIIT HV: 336	CRF, Fat mass, Fat-free mass, MSF
* Wilke et al., 2019 [17]	Healthy adults	WB-HIIT vs. MICT	WB-HIIT: Whole-body exercises MICT: Walking at 50–60% HRR	WB-HIIT: 30 MICT: 50	WB-HIIT: 20″/10″	WB-HIIT: 6 wks, 3×/wk MICT: 6 wks, 3×/wk	WB-HIIT: 540 MICT: 900	CRF, MSF
**Specific populations**								
Batrakoulis et al., 2018 [32]	Obese women	WB-HIIT vs. CTL	WB-HIIT: Whole-body exercises CTL: No exercise	WB-HIIT: 23–41	WB-HIIT: 20″/40″	WB-HIIT: 20 wks, 3×/wk	WB-HIIT: 1761	CRF, Fat mass, Fat-free mass, MSF
Jung et al., 2019 [33]	Women with sarcopenia	WB-HIIT vs. CTL	WB-HIIT: Whole-body exercises CTL: No exercise	WB-HIIT: 25–55	WB-HIIT: 10″/5″	WB-HIIT: 12 wks, 3×/wk	WB-HIIT: 1440	Fat mass, Fat-free mass
Scott et al., 2019 [34]	Obese adults	WB-HIIT vs. HIIT vs. MICT	WB-HIIT: Whole-body exercises at >80% theorical HRmax HIIT: Cycling at 100% Wmax MICT: Running/Cycling at 65% Theorical HRmax	WB-HIIT: 8–16 HIIT: 8–16 MICT: 30–50	WB-HIIT: 60″/60″ HIIT: 60″/60″	WB-HIIT: 12 wks, 3×/wk HIIT: 12 wks, 3×/wk MICT: 12 wks, 3×/wk	WB-HIIT: 432 HIIT: 432 MICT: 1440	CRF, Fat mass, Fat-free mass, Metabolic risk factors

CRF, cardiorespiratory fitness; CTL, control; HIIT, high-intensity interval training; HR, heart rate; HRR, heart rate reserve; HV, high volume; LV, low volume; MSF, musculoskeletal fitness; MICT, moderate-intensity continuous training; RPE, rate of perceived exertion; VICT, vigorous-intensity continuous training; VT2, second ventilatory threshold; WB-HIIT, whole-body high-intensity interval training; WB-MIIT, whole-body moderate intensity interval training; Wmax, maximal workload, wk(s), week(s), *, excluded from the meta-analysis.

**Table 2:** Narrative description of the intragroup changes after the intervention.

Study	Number of Participants	Male/Female	Age (Mean ± SD)	Cardiorespiratory Fitness	Body Composition	Musculoskeletal Fitness	Metabolic Risk Factors
**Healthy participants**							
Ballesta-Garcia, 2019 [11]	54	0/54	WB-HIIT: 66 ± 5 WB-MIIT: 70 ± 9 CTL: 67 ± 6	-	-	Arm curl (rep): WB-HIIT↑ WB-MIIT ↔ CTL↑ 30s sit to stand (rep): WB-HIIT↑ WB-MIIT ↑ CTL↓ Timed up and go (s) WB-HIIT↓ WB-MIIT ↓ CTL↑ One leg stand (left) (s): WB-HIIT↑ WB-MIIT ↔ CTL↔ One leg stand (right) (s): WB-HIIT↔ WB-MIIT ↔ CTL↔ Right handgrip (kg): WB-HIIT↔ WB-MIIT ↔ CTL↔ Left handgrip (kg): WB-HIIT↔ WB-MIIT ↔ CTL↔	-
Ballesta-Garcia, 2020 [12]	54	0/54	WB-HIIT: 66 ± 5 WB-MIIT: 70 ± 9 CTL: 67 ± 6	VO_2_max: WB-HIIT↑ WB-MIIT ↑ CTL↔	-	-	-
Blackwell et al., 2017 [20]	12	-	WB-HIIT: 52 ± 2 HIIT: 52 ± 3	VO_2_max: WB-HIIT↑ HIIT↑	-	-	SBP (mmHg): WB-HIIT↔ HIIT↔ DBP (mmHg): WB-HIIT↔ HIIT↔
* Connolly et al., 2020 [18]	24	0/24	WB-HIIT and CTL: 39 ± 10	-	-	-	SBP (mmHg): WB-HIIT↔ CTL↔ DBP (mmHg): WB-HIIT↔ CTL↔ Fasting glucose (mmol/L): WB-HIIT↔ CTL↔ HDL-C (mmol/L): WB-HIIT↑ CTL↔ LDL-C (mmol/L): WB-HIIT↔ CTL↔ Visceral fat (cm^3^): WB-HIIT↔ CTL↔
Engel et al., 2019 [22]	20	10/10	WB-HIIT: 35 ± 12 CTL: 37 ± 10	-	-	Leg press (rep): WB-HIIT↑ CTL↔ Chest press (rep): WB-HIIT↑ CTL↔ Pulldown (rep): WB-HIIT↑ CTL↔ Back extension (rep): WB-HIIT↔ CTL↔ Ventral plank (s): WB-HIIT↔ CTL↔ Left plank (s): WB-HIIT↑ CTL↔ Right plank (s): WB-HIIT↔ CTL↔	SBP (mmHg): WB-HIIT↔, CTL↔ DBP (mmHg): WB-HIIT↔ CTL↔
Evangelista et al., 2019 [23]	25	-	WB-HIIT: 28 ± 7 VICT: 29 ± 5	-	Fat mass (kg): WB-HIIT↔ VICT↔ Lean mass (kg): WB-HIIT↔ VICT↔	Abdominal (rep): WB-HIIT↔ VICT↔ Horizontal jump (min): WB-HIIT↔ VICT↔ Push-ups (rep): WB-HIIT↔ VICT↔	-
Evangelista et al., 2021 [24]	34	34/0	WB-HIIT: 28 ± 7 CTL: 29 ± 7	VO_2_max: WB-HIIT↑ CTL ↔	-	Push-ups (rep): WB-HIIT↑ CTL↔ Sit-ups (rep): WB-HIIT↑ CTL↔ Burpees: WB-HIIT↑ CTL↔ Leg press 1RM (kg): WB-HIIT↑ CTL↔	-
Islam et al., 2019 [25]	68	52/16	WB-HIIT: 21 ± 3 VICT: 22 ± 4 CTL: 21 ± 4	VO_2_max: WB-HIIT↔ VICT↑ CTL↔	-	Push-ups (rep): WB-HIIT↑ VICT↔ CTL↔ Right plank (s): WB-HIIT↑ VICT↔ CTL↔ Left plank (s): WB-HIIT↔ VICT↔ CTL↔ Back extension (s): WB-HIIT↔ VICT↔ CTL↔ Sit up (s): WB-HIIT↔ VICT↔ CTL↔	-
Jimenez-Garcia et al., 2019 [21]	73	20/62	WB-HIIT, WB-MIIT and CTL: 68 ± 3	-	Body fat (%): WB-HIIT↔ WB-MIIT↔ CTL↔ Muscle Mass (kg): WB-HIIT↔ WB-MIIT↔ CTL↔	Handgrip (kg): WB-HIIT↑ MIFT↔ CTL↔	-
Lu et al., 2021 [26]	20	0/20	WB-HIIT: 20 ± 1 HIIT: 21 ± 1	VO_2_max WB-HIIT ↑ HIIT ↑	Body fat (%): WB-HIIT↓ HIIT↓ Lean mass (kg): WB-HIIT↑ HIIT↑	Sit-ups (rep): WB-HIIT↑ HIIT↔ Push-ups (rep): WB-HIIT↔ HIIT↔ Broad jump (cm): WB-HIIT↑ HIIT↔	-
* McRae et al., 2012 [16]	22	0/22	WB-HIIT: 21 ± 1 MICT: 21 ± 3 CTL: 19 ± 1	VO_2_max: WB-HIIT↑ MICT↑ CTL↔	-	Leg extension (rep): WB-HIIT↑ MICT↔ CTL↔ Lateral pulldowns (rep): WB-HIIT↔ MICT↑ CTL↔ Chest press (rep): WB-HIIT↑ MICT↔ CTL↔ Push-ups (rep): WB-HIIT↑ MICT↔ CTL↔ Sit-ups (rep): WB-HIIT↑ MICT↔ CTL↔ Back extension (rep): WB-HIIT↑ MICT↔ CTL↔	-
Menz et al., 2019 [27]	15	4/11	WB-HIIT: 24 ± 2 HIIT: 27 ± 3	VO_2_max: WB-HIIT↑ HIIT↑	Body fat (%): WB-HIIT↔ HIIT↔ Muscle percentage (%): WB-HIIT↔ HIIT↔	Push-ups (rep): WB-HIIT↔ HIIT↔ Toes to bar (rep): WB-HIIT↑ HIIT↔ Burpees (rep): WB-HIIT↔ HIIT↑ Broad Jump (m): WB-HIIT↔ HIIT↔	-
Micielska et al., 2019 [28]	33	0/33	WB-HIIT and CTL: 38 ± 12	VO_2_max: WB-HIIT↑ CTL↔	Fat mass (kg): WB-HIIT↔, CTL↔ Muscle mass (kg): WB-HIIT↔ CTL↔	-	-
Murawska-Cialowicz et al., 2020 [29]	25	25/0	WB-HIIT: 32 ± 7 CTL: 25 ± 3	VO_2_max: WB-HIIT↑ CTL↔	Fat mass (kg): WB-HIIT↓ CTL↓ Muscle mass (kg): WB-HIIT↑ CTL↔	-	-
Schaun et al., 2018 [13]	41	41/0	WB-HIIT: 24 ± 2 HIIT: 23 ± 1 VICT: 24 ± 1	VO_2_max: WB-HIIT↑ HIIT↑ VICT↑	Body fat (%): WB-HIIT↓ HIIT↓ VICT↓	-	-
Schaun et al., 2019 [14]	41	41/0	WB-HIIT: 24 ± 2 HIIT: 23 ± 1 VICT: 24 ± 1	-	-	Counter movement jump height (cm): WB-HIIT↑ HIIT↑ VICT↑ Counter movement jump peak power (W): WB-HIIT↑ HIIT↑ VICT↔ Squat jump height (cm): WB-HIIT↑ HIIT↑ VICT↑ Squat jump peak power (W): WB-HIIT↑ HIIT↑ VICT↑	-
Schmidt et al., 2016 [30]	96	43/53	WB-HIIT 7 Male: 22 ± 2 WB-HIIT 14 Male: 21 ± 1 CTL Male: 21 ± 1 WB-HIIT 7 Female: 21 ± 1 WB-HIIT 14 Female: 21 ± 2 CTL Female: 20 ± 1	VO_2_max Male: WB-HIIT 7↔ WB-HIIT 14↔ CTL↔ VO_2_max Female: WB-HIIT 7↔ WB-HIIT 14↑ CTL↔	Body fat Male (%): WB-HIIT-7↔ WB-HIIT-14↔ CTL↔ Body fat Female (%): WB-HIIT-7↔ WB-HIIT-14↔ CTL↔	Right handgrip Male (kg): WB-HIIT 7↔ WB-HIIT 14↑ CTL↔ Left handgrip Male (kg): WB-HIIT-7↑ WB-HIIT-14↔ CTL↔ Push-ups Male (rep): WB-HIIT-7↑ WB-HIIT-14↑ CTL↔ Right handgrip Female (kg): WB-HIIT-7↔ WB-HIIT-14↔ CTL↔ Left handgrip Female (kg): WB-HIIT-7↔ WB-HIIT-14↔ CTL↔ Push-ups Female (rep): WB-HIIT-7↑ WB-HIIT-14↑ CTL↔	-
Sperlich et al., 2018 [31]	24	10/14	WB-HIIT-LV and -HV and CTL: 25 ± 5	VO_2_max: WB-HIIT-LV↔ WB-HIIT-HV↔ CTL↔	Fat mass (kg): WB-HIIT-LV↔ WB-HIIT-HV↔ CTL↔ Muscle percentage (%): WB-HIIT-LV↔ WB-HIIT-HV↔ CTL↔	Push-ups (rep): WB-HIIT-LV↑ WB-HIIT-HV↑ CTL↔ Leg-levers (rep): WB-HIIT-LV ↑ WB-HIIT-HV↑ CTL ↔ Burpees (rep): WB-HIIT- LV ↑ WB-HIIT-HV↑ CTL↔	-
* Wilke et al., 2019 [17]	33	12/21	WB-HIIT: 26 ± 6 MICT: 24 ± 3	VO_2_max: WB-HIIT↔ MICT↔	-	Leg press 1RM (kg): WB-HIIT↑ MICT↔ Chest press 1RM (kg): WB-HIIT↑ MICT↔ Single leg hop distance (cm): WB-HIIT↔ MICT↔ Counter movement jump (cm): WB-HIIT↔ MICT↔	-
**Specific populations**							
Batrakoulis et al., 2018 [32]	35	0/35	WB-HIIT: 36 ± 5 CTL: 36 ± 4	VO_2_max: WB-HIIT↑ CTL↔	Fat mass (kg): WB-HIIT↓ CTL↔ Fat-free mass (kg): WB-HIIT↑ CTL↔	Leg press 1RM (kg): WB-HIIT↑ CTL↔	-
Jung et al., 2019 [33]	26	0/26	WB-HIIT: 75 ± 4 CTL: 75 ± 5	-	Body fat (%): WB-HIIT↓ CTL↔ Fat-free mass (kg): WB-HIIT↑ CTL↔	-	-
Scott et al., 2019 [34]	32	13/19	WB-HIIT: 32 ± 8 HIIT: 37 ± 13 MICT: 38 ± 9	VO_2_max: WB-HIIT↑ HIIT↑ MICT↑	Body fat (%): WB-HIIT↓ HIIT↓ MICT↓ Lean mass (kg): WB-HIIT↔ HIIT↔ MICT↔	-	Fasting glucose (mmol/L): WB-HIIT↔ HIIT↔ MICT↔ HDL-C (mmol/L): WB-HIIT↔ HIIT↔ MICT↔ LDL-C (mmol/L): WB-HIIT↔ HIIT↔ MICT↔ SBP (mmHg): WB-HIIT↔ HIIT↔ MICT↔ DBP (mmHg): WB-HIIT↔ HIIT↔ MICT↔ Visceral fat (g): WB-HIIT↓ HIIT↓ MICT↓

CTL, control; DBP, diastolic blood pressure; HDL-C, high-density lipoproteins cholesterol; HIIT, high-intensity interval training; HV, high volume; LDL-C, low-density lipoproteins cholesterol; LV, low volume; MICT, moderate-intensity continuous training; 1RM, one-repetition maximum; SBP, systolic blood pressure; VICT, vigorous-intensity continuous training; WB-HIIT, whole-body high-intensity interval training; WB-MIIT, whole-body moderate intensity interval training; WB-HIIT 7, 7 min protocol; WB-HIIT 14, 14 min protocol; ↑, value increasing significantly; ↓, value decreasing significantly; ↔, no significant change. *, excluded from the meta-analysis.

**Table 3:** Methodological quality assessment (PEDro scale).

Studies	Inclusion Criteria	Random Allocation	Concealed Allocation	Groups Similar at Baseline	Blinded Participants	Blinded Therapist	Blinded Investigators	Data from >85% of Participants	Intention to Treat	Between Group Comparison	Estimation of Effect and Variability	TOTAL
Ballesta-Garcia, 2019 [11]	1	1	0	1	0	0	0	1	1	1	1	**6**
Ballesta-Garcia, 2020 [12]	1	1	0	1	0	0	0	1	1	1	1	**6**
Blackwell et al., 2017 [20]	1	1	0	1	0	0	1	1	1	1	1	**7**
Connolly et al., 2020 [18]	1	1	1	1	0	0	0	0	1	1	1	**6**
Engel et al., 2019 [22]	1	1	0	1	0	0	0	1	1	1	1	**6**
Evangelista et al., 2019 [23]	1	1	0	1	0	0	0	1	1	1	1	**6**
Evangelista et al., 2021 [24]	1	1	0	1	0	0	0	0	1	1	1	**5**
Islam et al., 2019 [25]	1	1	0	1	0	0	0	1	1	1	1	**6**
Jimenez-Garcia et al., 2019 [21]	1	1	1	1	0	0	0	1	1	1	1	**7**
Lu et al., 2021 [26]	0	1	0	1	0	0	0	1	1	1	1	**6**
McRae et al., 2012 [16]	0	0	0	1	0	0	0	1	0	1	1	**4**
Menz et al., 2019 [27]	1	1	0	1	0	0	0	0	1	1	1	**5**
Micielska et al., 2019 [28]	1	0	0	1	0	0	0	1	1	1	1	**5**
Murawska-Cialowicz et al., 2020 [29]	1	1	0	1	0	0	0	1	1	1	1	**6**
Schaun et al., 2018 [13]	1	1	0	1	0	0	0	1	1	1	1	**6**
Schaun et al., 2019 [14]	1	1	0	1	0	0	0	1	1	1	1	**6**
Schmidt et al., 2016 [30]	1	1	0	1	0	0	0	1	1	1	1	**6**
Sperlich et al., 2018 [31]	1	1	0	1	0	0	0	1	1	1	1	**6**
Wilke et al., 2019 [17]	1	1	0	1	0	0	0	1	1	1	1	**6**
Batrakoulis et al., 2018 [32]	1	1	0	1	0	0	0	1	1	1	1	**6**
Jung et al., 2019 [33]	1	1	0	1	0	0	0	1	1	1	1	**6**
Scott et al., 2019 [34]	1	0	0	1	0	0	0	1	1	1	1	**5**

### 3.3. Characteristics of the Included Studies

Among the nineteen studies included in the meta-analysis, sixteen investigated the impact of WB-HIIT in healthy participants [11,12,13,14,20,21,22,23,24,25,26,27,28,29,30,31] and three in populations with physical limitations or metabolic risk factors [32,33,34] (two in obese adults and one in women with sarcopenia). Eleven studies [11,12,21,22,24,28,29,30,31,32,33] compared WB-HIIT to a no exercise control group only, seven studies [13,14,20,23,26,27,34] had a traditional aerobic training comparator group, and one study [25] included both. Thirteen studies [12,13,20,24,25,26,27,28,29,30,31,32,34] assessed CRF, twelve [13,21,22,26,27,28,29,30,31,32,33,34] assessed fat mass, ten [21,23,26,27,28,29,31,32,33,34] assessed fat-free mass, and twelve [11,14,21,22,23,24,25,26,27,30,31,32] assessed musculoskeletal fitness.

### 3.4. WB-HIIT Compared to No Exercise

**Figure 2:**
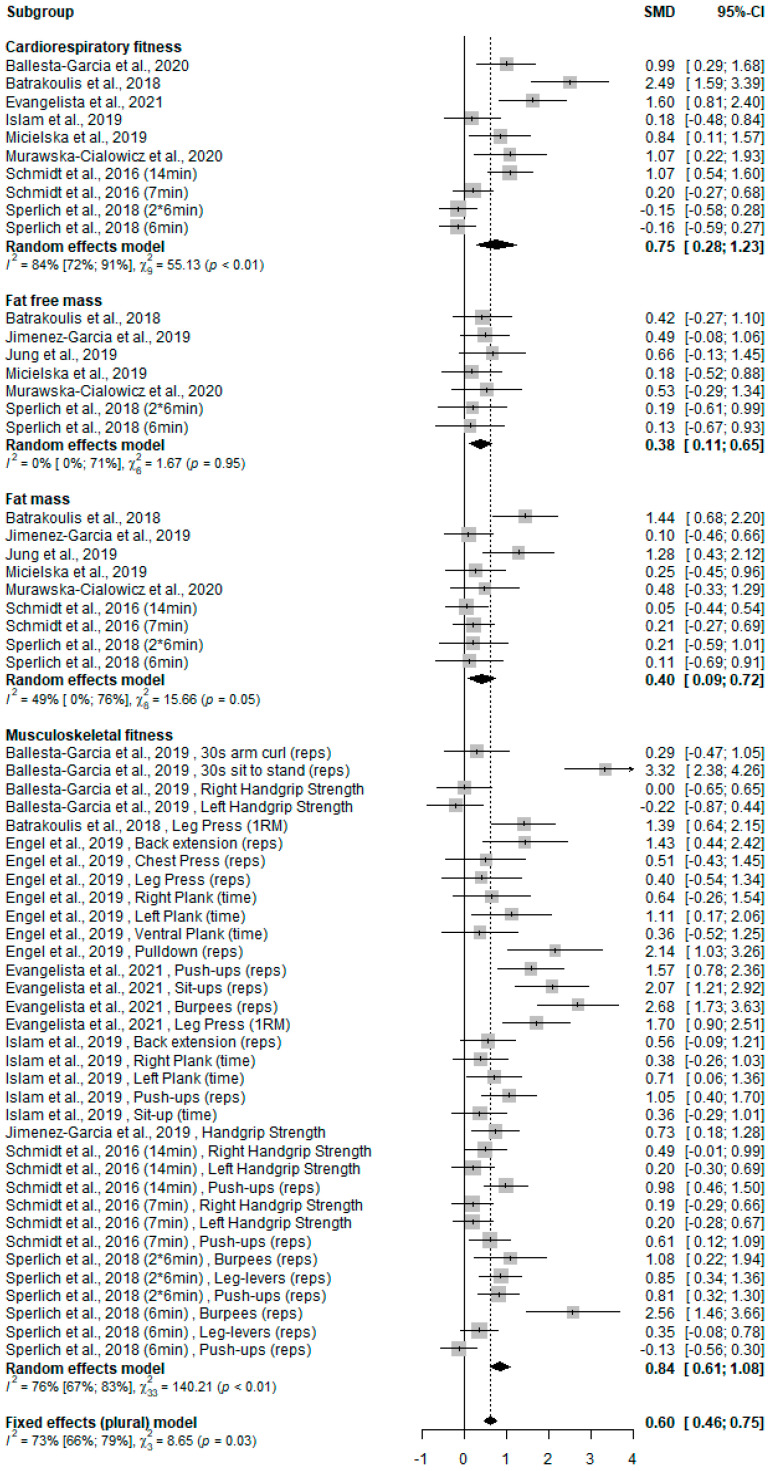
Forest plot of standardized mean difference (SMD) and confidence interval (CI) for the comparison WB-HIIT vs. no exercise. Positive values favor WB-HIIT. Subgroups mean effect and overall effect are written in bold. Reps, number of repetitions performed during a certain amount of time or until exhaustion; 1RM, one-repetition maximum.

Of the twelve studies [11,12,21,22,24,25,28,29,30,31,32,33] with a no exercise comparator, eight [12,24,25,28,29,30,31,32] evaluated CRF, six [21,28,29,31,32,33] changes in fat-free mass, seven [21,28,29,30,31,32,33] changes in fat mass, and eight [11,21,22,24,25,30,31,32] musculoskeletal fitness. The overall effect on health-related fitness was significantly higher for WB-HIIT (SMD: 0.60, 95% CI: 0.46 to 0.75; *p* < 0.001). When analyzing the different components, WB-HIIT statistically improved CRF (SMD: 0.75, 95% CI: 0.28 to 1.23; *p* < 0.001), fat-free mass (SMD: 0.38, 95% CI: 0.11 to 0.65; *p* < 0.001), fat mass (SMD: 0.40, 95% CI: 0.09 to 0.72; *p* < 0.001) and musculoskeletal fitness (SMD: 0.84, 95% CI: 0.61 to 1.08; *p* < 0.001). The forest plot summarizing the results is presented in Figure 2.

No meta-analysis was carried out for metabolic risk factors since only two studies assessed those [18,22]. They observed no change in blood pressure, fasting glucose, visceral fat or LDL cholesterol, and an increase in HDL cholesterol in the WB-HIIT group (Table 2) [18,22].

### 3.5. WB-HIIT vs. Traditional Aerobic Training

**Figure 3:**
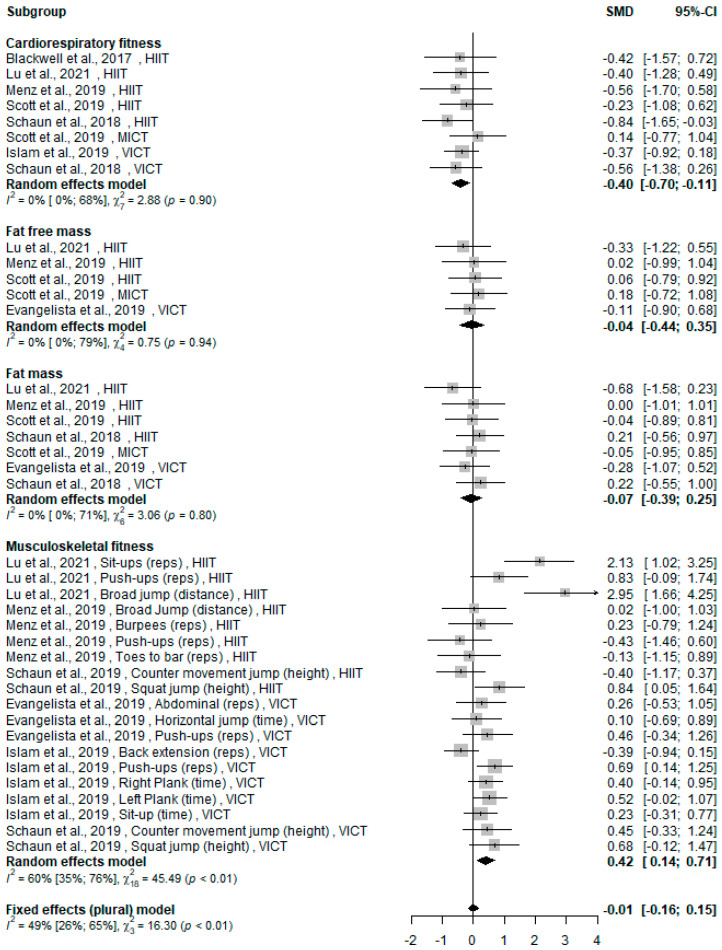
Forest plot of standardized mean difference (SMD) and confidence interval (CI) for the comparison WB-HIIT vs. traditional aerobic training. Positive values favor WB-HIIT. Subgroups mean effect and overall effect are written in bold. HIIT, high-intensity interval training; MICT, moderate-intensity continuous training; VICT, vigorous-intensity continuous training; Reps, number of repetitions performed during a certain amount of time or until exhaustion.

Of the eight [13,14,20,23,25,26,27,34] studies comparing WB-HIIT to traditional aerobic training, three [13,14,34] included two forms of training as comparator. All traditional aerobic training modalities (HIIT, MICT or VICT) were gathered in the same forest plot (Figure 3). Six studies [13,20,25,26,27,34] assessed CRF, four [23,26,27,34] fat-free mass, five [13,23,26,27,34] fat mass and five [14,23,25,26,27] musculoskeletal fitness.

The overall effect on health-related fitness of WB-HIIT was not statistically different from traditional aerobic training (SMD: −0.01, 95% CI: −0.16 to 0.15; *p* = 0.22). Regarding CRF, a small effect was observed in favor of traditional aerobic training (SMD: −0.40, 95% CI: −0.70 to −0.11; *p* = 0.007). In contrast, a small effect was observed in favor of WB-HIIT for musculoskeletal fitness (SMD: 0.42; 95% CI: 0.14 to 0.71; *p* = 0.003). No difference was found between WB-HIIT and other training modalities for fat-free mass (SMD: −0.04, 95% CI: −0.44 to 0.35; *p* = 0.8) or fat mass (SMD: −0.07, 95% CI: −0.39 to 0.25; *p* = 0.7). The forest plot summarizing the results is presented in Figure 3.

The two studies investigating metabolic risk factors observed no effect on blood pressure, fasting glucose, HDL or LDL cholesterol, whatever the training modality (Table 2) [20,34]. One study observed a similar decrease in visceral fat in WB-HIIT and traditional aerobic trainings [34].

### 3.6. Total Training Time

**Figure 4:**
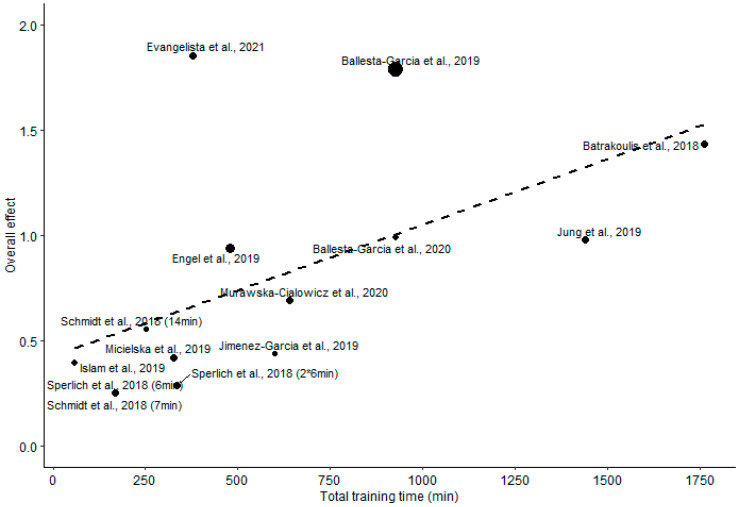
Regression between the total training time and the overall effect of WB-HIIT. Filled circles size is proportional to the study weight.

A wide interstudy variability of WB-HIIT total training time was observed, ranging from 48 to 1761 min. The meta-regression analysis showed that overall effect of WB-HIIT on health-related fitness is associated (*p* = 0.004) with total training time (Figure 4 and Table 4). A significant association was also found for the effect on CRF (*p* = 0.001) and fat mass (*p* < 0.001), but not for fat-free mass and musculoskeletal fitness (*p* = 0.41 and 0.1, respectively; Table 4).

## 4. Discussion

We conducted a systematic review and meta-analysis to assess the efficacy of WB-HIIT to improve health-related fitness as compared to no exercise or traditional forms of aerobic training. The present results show that WB-HIIT is effective in improving CRF, fat-free mass, fat mass and musculoskeletal fitness when compared to no exercise. WB-HIIT is less effective than traditional forms of aerobic training for enhancing CRF but is equally effective for improving fat-free mass and fat mass. The meta-analysis highlights a higher effect of WB-HIIT on musculoskeletal fitness compared to other forms of aerobic training. The overall effect of WB-HIIT on health-related fitness, as well as specific effects on CRF and fat mas, is associated with the total training time, indicating the existence of a dose-response relationship. The absence of a significant association between fat-free mass and musculoskeletal fitness suggests that other parameters (e.g., exercises type, load, motor skills trained, etc.) are predominant in improving these components.

### 4.1. Cardiorespiratory Fitness

Since the WB-HIIT protocols included in the meta-analysis were designed to reach high intensities during the exercise phases, the greater effect of WB-HIIT on CRF, compared to no exercise, is consistent with previous findings [5]. A novel observation is the existence of a dose–response relationship supported by the regression between the total training time of WB-HIIT and the effect on CRF.

When compared to traditional forms of aerobic training, our results show a lower effect of WB-HIIT to improve CRF. The lower effectiveness as compared to VICT could be explained by the shorter total training time in WB-HIIT and the high intensity of VICT, resulting in a greater training volume (time × intensity) for the latter. This hypothesis is supported by the observation that the study that compared WB-HIIT to MICT, characterized by a lower intensity than VICT, showed similar improvement after both training types [34]. The HIIT protocols included in the present meta-analysis were mostly time equivalent to WB-HIIT. Therefore, total training time alone could not explain the greater improvement of CRF. A potential explanation is that WB-HIIT protocols fail to reach an intensity as high as traditional forms of HIIT, which would result in a smaller improvement of VO_2_max [35,36]. Target intensity for WB-HIIT is often mentioned as “all out”, while it is described as a percentage of maximal HR or VO_2_max in HIIT protocols. Only one study reported peak and mean HR reached during training sessions, and they were lower for WB-HIIT compared to HIIT [27]. The findings highlight the importance of monitoring training intensity to provide comparable data in the future.

### 4.2. Fat Mass and Fat-Free Mass

Compared to no exercise, the meta-analysis indicates a significant decrease in fat mass for WB-HIIT. The impact of total training time on fat mass loss supports the existence of a dose–response relationship. This finding is consistent with previous observations showing that HIIT with a sufficient energy expenditure is required to reduce body fat mass [37].

When comparing WB-HIIT to traditional forms of aerobic training, the effect on fat mass is not different. As mentioned above, the intensity reached during WB-HIIT protocols was probably lower compared to HIIT and VICT, resulting in a lower training volume. Therefore, the similar effect on fat mass could at first be surprising. However, compared to traditional HIIT and VICT, WB-HIIT induces a greater muscle mass recruitment through exercises involving lower and upper limbs and core muscles, which would increase energy expenditure despite a lower training intensity. Unfortunately, no study compared energy expenditure between traditional aerobic trainings and WB-HIIT protocols. Nevertheless, WB-HIIT appears to be as effective as traditional aerobic training to decrease body fat mass when the amount of training is sufficient.

Although resistance training using high loads is generally considered as the gold standard to induce muscle hypertrophy, emerging evidence suggests that interval training, such as HIIT or sprint interval training, has the potential to upregulate cellular mechanisms underlying lean mass increases [38]. The enhancement of fat-free mass by WB-HIIT supports this observation. Similar to HIIT, WB-HIIT involves high execution speed and short resting periods but also stretch-shortening cycles that favor recruitment of type 2 muscle fibers and thereby promote muscle hypertrophy [38].

The effect of WB-HITT on fat-free mass was not different from traditional forms of aerobic training. The selected WB-HIIT and traditional aerobic training protocols were designed to elicit cardiovascular impact and were not optimized to improve muscle mass. This could explain the low effect on fat-free mass [23,27,34] for WB-HIIT as well as for traditional forms of aerobic training (Table 2). One advantage of WB-HIIT over traditional HIIT is the possibility to enhance muscle recruitment by selecting adequate exercises and/or adding free weights.

### 4.3. Musculoskeletal Fitness

Our results revealed that WB-HIIT is more effective than no exercise and traditional aerobic trainings to improve musculoskeletal fitness. To assess musculoskeletal fitness, strength tests (e.g., handgrip and one-repetition maximum) and muscular endurance tests (e.g., planks, sit-ups, push-ups, squat jumps, etc.) were used. Improvements in strength tests are known for being mainly related to greater muscle recruitment, [39] while muscular endurance tests involve muscle endurance and strength but also segmental coordination, core stability and/or explosiveness. Since muscular endurance tests include motor skills/coordination needed for execution of daily tasks (e.g., pulling, pushing, squat, etc.), they are considered to better reflect functional abilities than usual strength tests [40,41].

The greater improvement in musculoskeletal fitness after WB-HIIT seems intrinsically linked to the training modality. Traditional forms of aerobic training are generally unimodal (e.g., running, rowing, cycling, etc.), while WB-HIIT is multimodal [2] and involves specific motor skills and core stability that are necessary for the performance of muscular endurance tests and multi-joint dynamic strength tests. This is supported by our results showing larger increases after WB-HIIT in the number of repetitions performed during dynamic endurance tests [16,22,24,25,26,30] or functional strength tests [11,31] and in leg press one-repetition maximum [17,24,32], compared to no exercise [11,16,22,24,25,30,31,32] or traditional aerobic trainings [16,17,25,26], while a lower effect for isometric strength tests was observed [11,30]. Based on the concept of motor skills transfer [42], WB-HIIT hence appears more effective for improving functional musculoskeletal fitness than traditional aerobic training.

### 4.4. Metabolic Risk Factors

Only two studies assessed metabolic risk factors after WB-HIIT vs. no exercise, and two compared WB-HIIT to other aerobic training. Except for the increased HDL levels with WB-HIIT in one study [18], and the decreased visceral fat in another study [34], no changes were found for blood pressure or for fasting blood glucose or lipids, regardless of training type [18,20,22,34]. In all four studies, these variables were within normal ranges and were therefore less likely to improve. Studies investigating the effect of WB-HIIT in patients with metabolic syndrome are therefore needed.

### 4.5. Limitations and Recommendations

There are some limitations to this meta-analysis. First, session number and frequency and intervention duration differed greatly between studies. Only one study [27] recorded intensity attained during sessions, precluding between study comparisons of total training volume that can greatly impact CRF and body composition [37]. Second, a large variety of tests were used to assess musculoskeletal fitness, explaining the variability in observed effects (see Figure 2 and Figure 3), and complicating study comparisons. Future studies should systematically monitor training intensity, compare WB-HIIT to traditional aerobic trainings of similar volume and include standardized tests to assess muscle strength and endurance. Lastly, only few studies used WB-HIIT in participants with physical limitations (one study on sarcopenia [33]) and metabolic risk factors (two studies including obese subjects [32,34]), or assessed effects of WB-HIIT on lipid profile, blood pressure or fasting glucose level [18,20,22,34]. Visceral fat, an important cardiovascular disease risk factor, was measured in only one study comparing WB-HIIT to no exercise [18] and one comparing WB-HIIT to traditional training [34]. Given this paucity of data, the impact of WB-HIIT on cardio-metabolic risk factors can currently not be meta-analyzed. Further studies are needed to investigate the potential of WB-HIIT, especially in patients with established risk factors.

Some practical recommendations could be formulated based on our results. Since the effect of WB-HIIT appears to be related to total training time, and because previous studies have reported that intensity influences body composition and CRF changes [4,37,43], WB-HIIT practitioners should ensure that training duration and exercise intensity attained during the active phases is sufficient to induce significant improvements. As discussed above, WB-HIIT is characterized by fast and explosive multi-joint exercises involving more functional motor skills and segmental coordination than traditional aerobic trainings. WB-HIIT should therefore be incorporated into physical training programs to facilitate transfer to daily activities and sports requiring multi-joint movements. Traditional HIIT and WB-HIIT both favor type 2 fibers recruitment [38] known to foster muscle mass and muscle performance increase. Additional studies comparing different protocols of WB-HIIT and HIIT would help to understand the determinants of training-related adaptations. This step is essential to advise specific training characteristics maximizing body composition and musculoskeletal fitness improvements, depending on the context and target population.

## 5. Conclusions

WB-HIIT is a low-cost, easy to implement, and effective way for improving CRF, body composition and musculoskeletal fitness. Although traditional forms of aerobic training are more effective at improving CRF, WB-HIIT induces equivalent improvements in body composition and has greater effects on musculoskeletal fitness required for execution of daily tasks. Importantly, our results highlight the dose–response relationship between WB-HIIT effects on CRF and fat mass and total training time. Since intensity affects training-related changes, it is essential that future studies systematically assess this parameter. Further studies are needed to establish if WB-HIIT improves autonomy and cardio-metabolic health in patients with physical limitations and metabolic risk factors, and to define the most effective WB-HITT protocols to improve specific components of health-related fitness.

## Figures and Tables

**Table 4:** Association between total training time of WB-HIIT and overall effect or effect on individual components (per hour of training).

Parameters	K	Beta (SE)	*p* Value
Overall effect	14	0.036 (0.012)	0.004
Cardiorespiratory fitness	10	0.078 (0.024)	0.001
Fat-free mass	7	0.012 (0.018)	0.41
Fat mass	9	0.048 (0.012)	<0.001
Musculoskeletal fitness	10	0.030 (0.018)	0.1

K, number of included studies; Beta, regression beta coefficients; SE, standard error.

## Data Availability

All the data and codes used in the conduction of this meta-analysis are available from the corresponding author upon reasonable request.

## Supplementary Materials

The following supporting information can be downloaded at: www.mdpi.com/article/10.3390/ijerph19159559/s1, PRISMA_2020_checklist IJERPH submission [44]; Supplementary Figure S1 Funnel Noexercise; Supplementary Figure S2 Funnel Active; Supplementary Figure S3 Sensitivity Noexercise; Supplementary Figure S4 Sensitivity Active.
