# Supplementary material for: Effectiveness of Whole-Body High-Intensity Interval Training on Health-Related Fitness: A Systematic Review and Meta-Analysis
Source: Int J Environ Res Public Health. 2022 Aug 3;19(15):9559. doi: 10.3390/ijerph19159559 (PMC9367756; doi:10.3390/ijerph19159559)
Supplement: Supplementary file 1 [file ijerph-19-09559-s001.zip › Supplementary Figures.pdf]

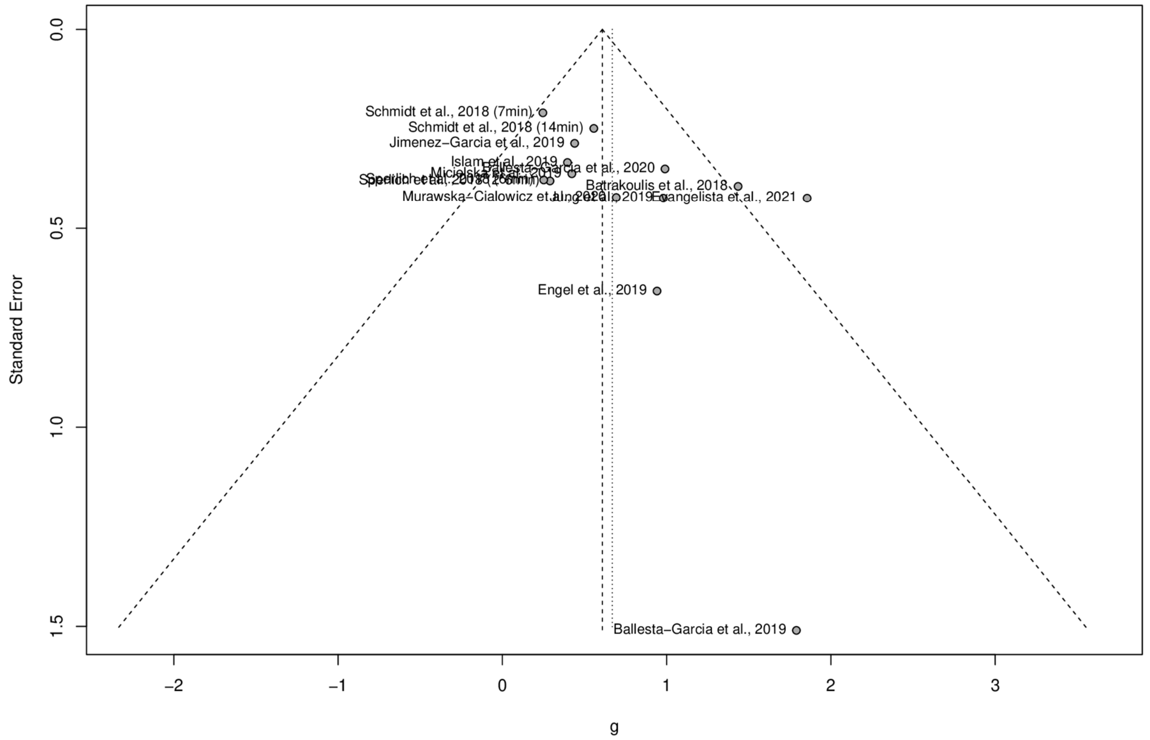

Figure S1. funnel noexercise

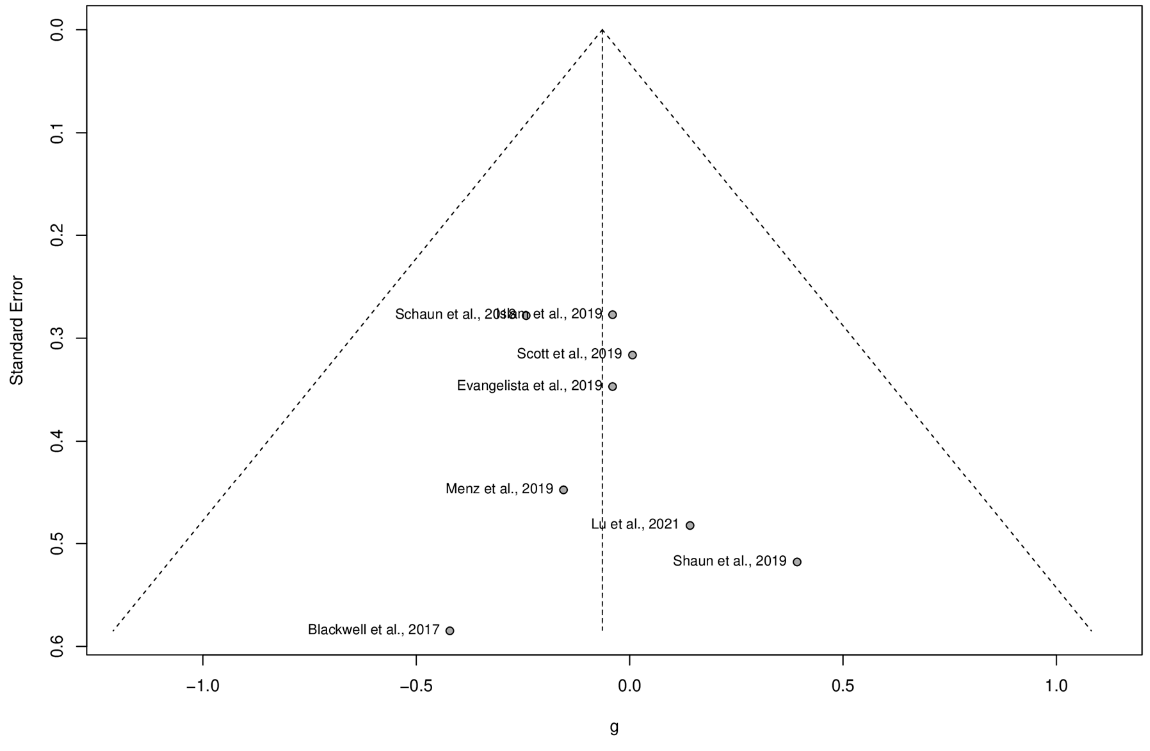

Figure S2. funnel active

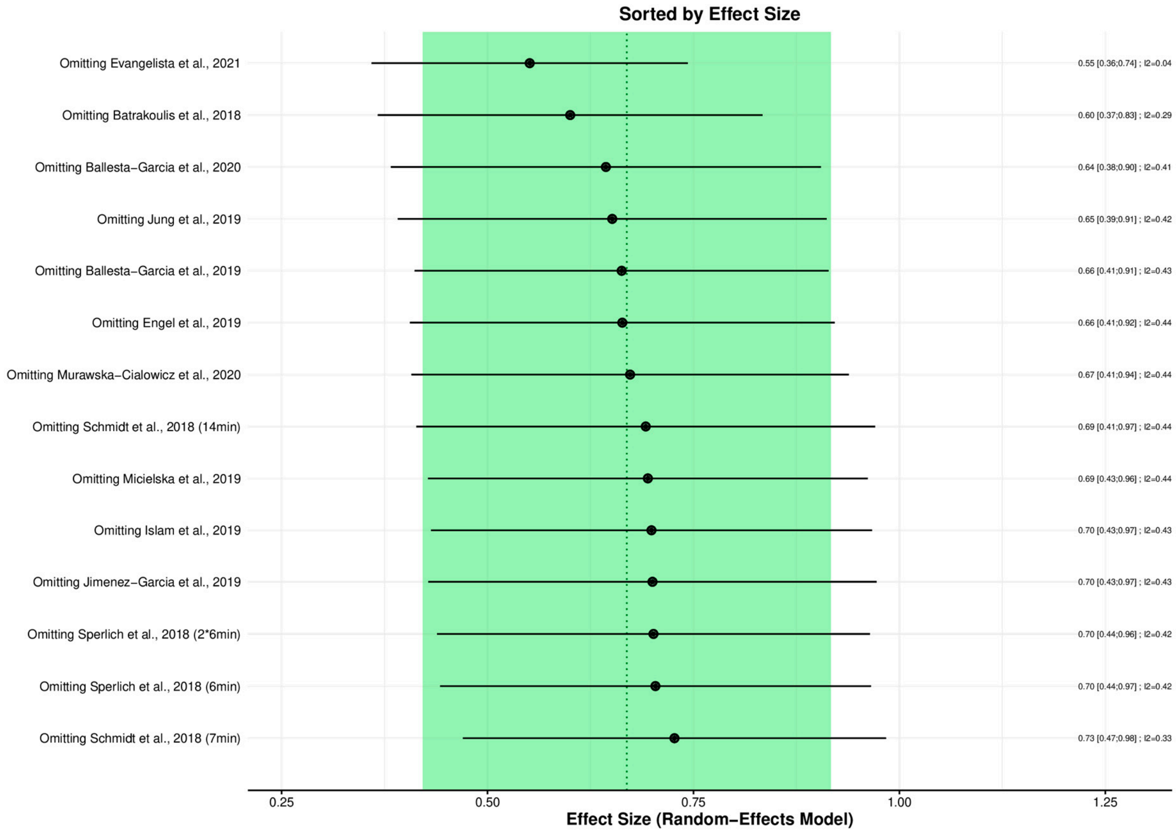

Figure S3. sensitivity noexercise

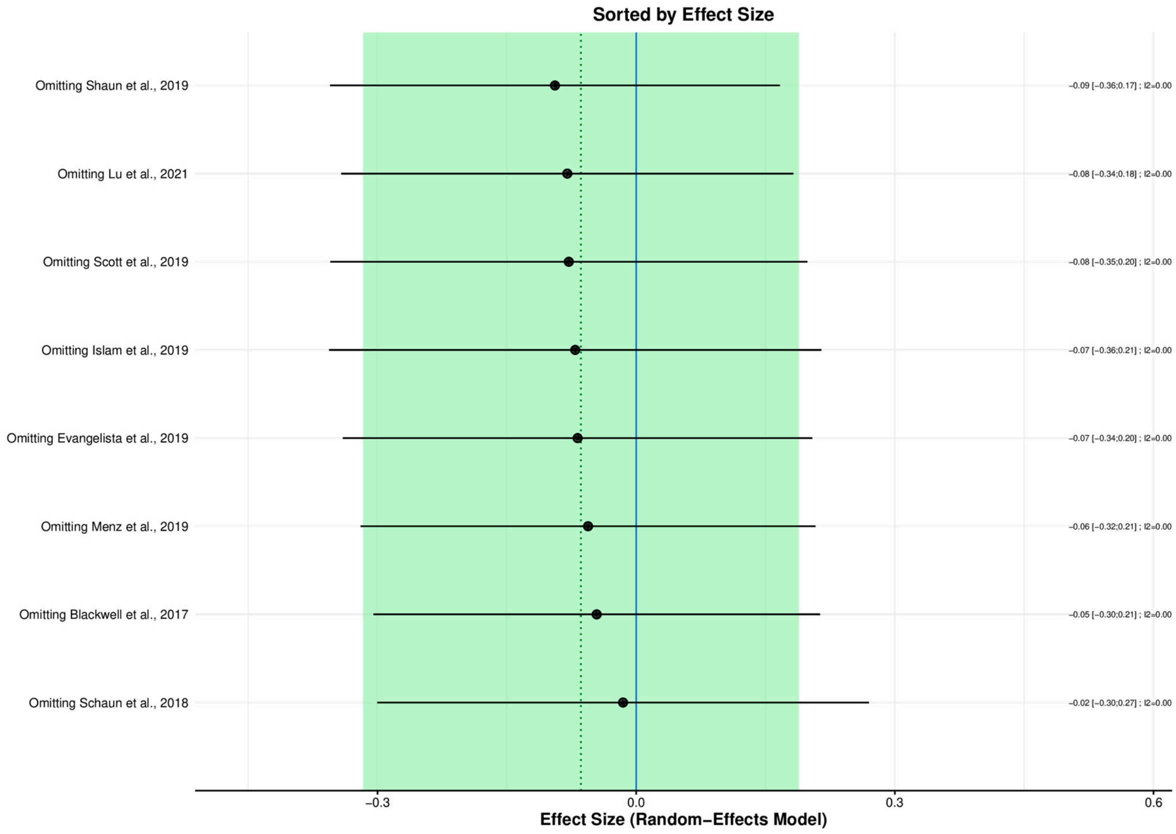

Figure S4. sensitivty active
